# Non-destructive testing of interfacial stiffness based on spring model for diffusion bonding interface of titanium alloy components with complex surface

**DOI:** 10.1038/s41598-023-42887-4

**Published:** 2023-09-21

**Authors:** Gongpeng Yang, Zhenggan Zhou, Tengfei Ma, Lichen Teng, Jun Wang, Yuxuan Zhou, Yang Li, Wenbin Zhou

**Affiliations:** 1 School of Mechanical Engineering and Automation, Beihang University, Beijing, 100191 China; 2 School of Energy and Power Engineering, Beihang University, Beijing, 100191 China; 3 Ningbo Institute of Technology, Beihang University, Ningbo, 315800 China

**Keywords:** Aerospace engineering, Engineering, Mechanical engineering

## Abstract

Ultrasonic testing is an important non-destructive testing method, which is sensitive to the defects in the diffusion bonding interface. Ultrasonic testing of diffusion bonding interfaces in complex-surface components is a challenge due to the geometry and the weak echo signal of the diffusion bonding defects. This paper proposes an interfacial stiffness characterization method based on the spring model for the ultrasonic testing of the diffusion bonding interface of titanium alloy complex-surface component. Finite element models for ultrasonic field are established to analyze the diffusion bonding defects response, the effect of complex surface, and the inconsistency of the bonding interface depth in ultrasonic testing of the titanium alloy complex-surface component. 15 MHz is recommended as the testing frequency of the diffusion bonding interface. Ultrasonic C-scan experiments are conducted using specimens with embedded artificial defects and a titanium alloy complex-surface component. The simulation and experimental results show that the novel interfacial stiffness characterization method can be applied to ultrasonic testing of the diffusion bonding interface (inclination angle less than 14°) in complex-surface components, and the ability to test defects at the diffusion bonding interface can be improved.

## Introduction

Diffusion bonding is a solid-state welding method connecting the same or different materials through atomic diffusion at specific temperatures and pressure in a vacuum environment^1^. The diffusion bonding joint has mechanical properties close to the base metal^2,3^, high bonding accuracy, and minor deformation, widely used in complex aerospace components such as aero-engine blades^4,5^. Affected by the bonding process and the surface condition of materials^6,7^, the interface of diffusion bonding is prone to defects such as lack of bonding, voids, and even tiny pores, seriously threating its service performance^8–11^. It is necessary to use non-destructive testing (NDT) methods to evaluate the bonding quality.

Ultrasonic testing has the advantages of strong penetration and no harm to the human, and it is more sensitive to interface defects than NDT methods such as X-ray and the eddy current. When incomplete fusion occurs after diffusion bonding, tiny pores defects are distributed on the diffusion bonding interface, whose sizes can be at the micron scale and much lower than the conventional ultrasonic wavelength (wavelength above 0.4 mm, frequency below 15 MHz), resulting in weak echo signals of such defects^12^. In addition, when the testing object has a complex-surface structure, its shape and geometric characteristics of the internal diffusion bonding interface challenge the reception of weak ultrasonic echo signals from diffusion bonding defects.

For ultrasonic testing of diffusion bonding interface, high-frequency (frequency above 20 MHz) ultrasonic testing^13^ or nonlinear ultrasonic method^14^ is commonly used. To characterize imperfect interfaces such as the diffusion bonding interface, Baik et al.^15–17^ proposed a spring model to equivalent the interfaces. The diffusion bonding interface is regarded as a thin elastic layer composed of a series of discrete springs with mass. This model has been widely used in ultrasonic testing for the coating/substrate structure^18^, oil layers^19,20^, adhesive joints^21–23^, austenitic weld interface^24^, composite laminates^25^, etc. Escobar et al.^26,27^ combined the signal true phase measurement with the spring model of ultrasonic reflection at the diffusion bonding interface. They used the interfacial stiffness *ĸ* calculated from the true phase of the diffusion bonding echo to characterize the bonding quality of the titanium alloy diffusion bonding interface.

For ultrasonic testing of complex-surface components, many methods have been proposed to solve the problem of obtaining the morphology of the incident surface of ultrasonic waves, including manual profiling of the ultrasonic transducer^28,29^, laser profiling^30^, digital-analog importing^31^, and adaptive methods of the array transducer^32,33^. Among these methods, laser profiling has the characteristics of high efficiency and accuracy. It is suitable for the geometric information extraction of the incident surface of the complex-surface components with unknown models.

This paper proposes an interfacial stiffness characterization method based on spring model for ultrasonic testing of the diffusion bonding interface of titanium alloy complex-surface components. The model of diffusion bonding interface in complex surface components is established considering: the defect response, influence of the complex surface, and the varying depth of bonding interface. Dynamic and static finite element (FE) models of ultrasonic field are established. Through these models, the validity of the interfacial stiffness method based on the defects phase is verified. The influence of noise on the phase measurement of defect echo in diffusion bonding at different frequencies, the curvature of the incident surface, and the inclination angle of the bonding interface on the phase measurement of diffusion bonding are analyzed. For the complex-surface component, an experiment integrating laser profiling and ultrasonic testing is carried out on a self-developed robot testing system.

## Ultrasonic reflection of diffusion bonding interface

The diffusion bonding interface is equivalently characterized by a thin elastic layer. The spring model with interfacial stiffness *ĸ* is expressed as Eq. (1)^15,16,20^:

1 $$ R_{12} = \frac{{\left( {\frac{{Z_{2} - Z_{1} }}{{Z_{1} + Z_{2} }}} \right)\left( {1 - \frac{{m\omega^{2} }}{4\kappa }} \right) + i\omega \left[ {\frac{{Z_{1} Z_{2} }}{{(Z_{1} + Z_{2} )\kappa }} - \frac{m}{{Z_{1} + Z_{2} }}} \right]}}{{\left( {1 - \frac{{m\omega^{2} }}{4\kappa }} \right) + i\omega \left[ {\frac{{Z_{1} Z_{2} }}{{(Z_{1} + Z_{2} )\kappa }} + \frac{m}{{Z_{1} + Z_{2} }}} \right]}} $$

where *R*_12_ is the reflection coefficient of the interface, *Z*_1_ and *Z*_2_ are the acoustic impedance of the materials on sides of the interface, *m* is the mass of tiny pores per unit area, *ω* is the angular frequency, and *ĸ* is the interfacial stiffness per unit length. After the diffusion bonding, the acoustic impedance of the interface changes. For the both sides of interface made of the TC4 titanium, the harmonic mean acoustic impedance *Z* and the relative acoustic impedance mismatch *η* are introduced as Eq. (2)^34^:

2 $$ \left\{ \begin{gathered} Z = \frac{{2Z_{1} Z_{2} }}{{Z_{1} + Z_{2} }} \hfill \\ \eta = \frac{{Z_{2} - Z_{1} }}{{Z_{1} + Z_{2} }} \hfill \\ \end{gathered} \right. $$

Diffusion bonding defects are arrays of tiny pores with mass *m* ≈ 0. By substituting Eq. (2) into Eq. (1), the reflection coefficient of titanium alloy diffusion bonding interface *R*_bond_ can be obtained as Eq. (3):

3 $$ R_{{{\text{bond}}}} = \frac{{\eta + \frac{i\omega Z}{{2\kappa }}}}{{1 + \frac{i\omega Z}{{2\kappa }}}} $$

Im(*R*_bond_) and Re(*R*_bond_) are the imaginary and real parts of the reflection coefficient *R*_bond_ respectively. The phase shift Φ_bond_ of the reflected signal generated by the diffusion bonding interface is as Eq. (4):

4 $$ \Phi_{{{\text{bond}}}} = \arctan \left( {\frac{{{\text{Im}} \left( {R_{{{\text{bond}}}} } \right)}}{{{\text{Re}} \left( {R_{{{\text{bond}}}} } \right)}}} \right) $$

It can be assumed *ωZ* < *ĸ* due to diffusion bonding interface characteristics^34^. Then the real and imaginary parts of the reflection coefficient *R*_bond_ can be approximated to Re(*R*_bond_) ≈ *η* and Im(*R*_bond_) ≈ *ωZ* / 2*ĸ*. The interfacial stiffness *ĸ* of the diffusion bonding interface can be calculated from the measured Φ_bond_ as Eq. (5):

5 $$ \kappa \approx \left| {\frac{\omega Z}{{2\eta \tan \left( {\Phi_{{{\text{bond}}}} } \right)}}} \right| $$

According to Eq. (5), the interfacial quality of diffusion bonding can be evaluated as interfacial stiffness *ĸ*. *η* can be calculated as Eq. (6):

6 $$ \eta = \frac{{\left| {H_{{{\text{bond}}}} \left( f \right)} \right|}}{{\left| {H_{{{\text{refer}}}} \left( f \right)} \right|}} $$

where |*H*_bond_(*f*)| and |*H*_refer_(*f*)| are the amplitude of the echo signal from bonding interface and the reference echo signal from titanium-water interface collected before diffusion bonding at the center frequency *f* of the ultrasonic transducer, respectively^27^. A portion of the phase spectrum *φ*(*ω*) of the pulse echo signal around the center angular frequency *ω*_0_ can be approximated as a fitting-line. The phase value at the intersection of the above line and zero-frequency axis is independent of the arrival time of the pulse and is named as the "true phase" *φ*_true_ as Eq. (7)^35,36^:

7 $$ \varphi \left( \omega \right) = \varphi_{{{\text{true}}}} + \beta \omega \, \left( {\varphi_{{{\text{true}}}} = \varphi_{{\text{true - cal}}} ,\varphi_{{\text{true - refer}}} } \right) $$

where *β* is the slope of the best fitting-line around the center frequency. The true phase *φ*_true_ is the value of the intersection between the tangent of the phase spectrum *φ*(*ω*) at the center frequency *f* and the zero-frequency axis. The phase shift caused by the diffusion bonding interface can be calculated from the true phase of the calculated signal *φ*_true-cal_ and the true phase of the reference signal *φ*_true-refer_ as Eq. (8):

8 $$ \Phi_{{{\text{bond}}}} = \varphi_{{\text{true - refer}}} - \varphi_{{\text{true - cal}}} + \frac{\pi }{2} $$

## Ultrasonic testing simulation model

In terms of the practical application of ultrasonic testing for defects on the diffusion bonding interface of the complex-surface component, the FE model of micron-scale pores defect testing, the FE model of ultrasonic testing considering the curvature of the incident surface and the inclination angle of the testing interface, and the static ultrasonic field computation model considering the coverage of bonding interface with a specific range of depth are established.

### Ultrasonic response model of diffusion bonding defects

**Figure 1:**
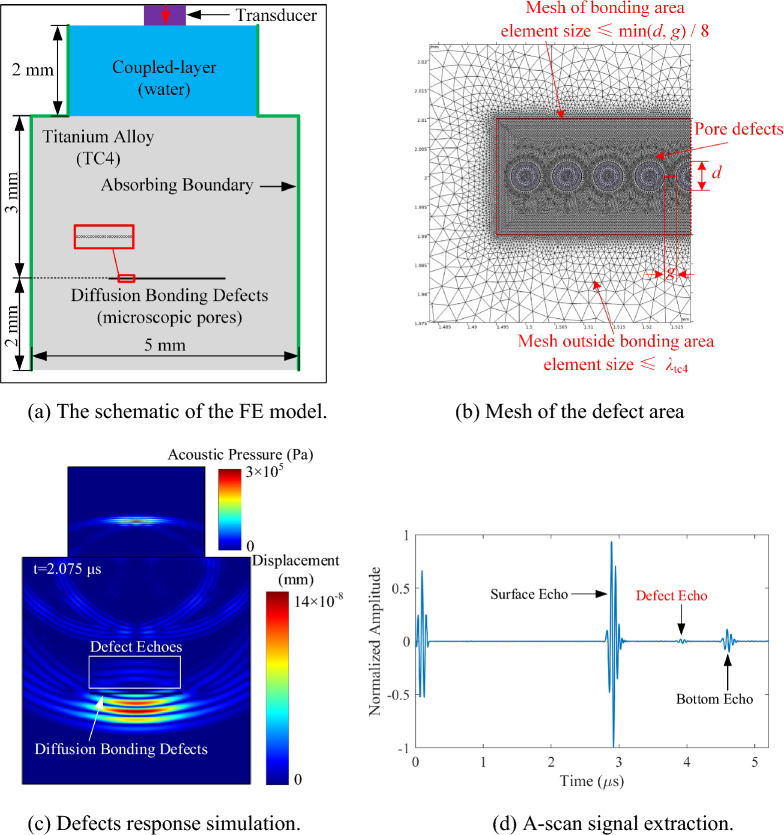
FE model of diffusion bonding microscopic pores defects.

Diffusion bonding defects are arrays of tiny pores distributed on the bonding interface in micron level. In this paper, diffusion bonding defects are modeled using an array of the pores with uniform size. Parameters of diffusion bonding defects are simplified to be the bonding rate and the pore size. The bonding rate represents the rate of the good bonding length to the total bonding length in the 2D FE model, expressed as Eq. (9):

9 $$ r = g/\left( {d + g} \right) $$

where *d* is the pore diameter and *g* is the spacing distance between the pore in the tiny pores array. The ultrasonic testing configuration, such as the ultrasonic transducer, the water layer, TC4 titanium alloy material, and absorption layers, are defined in the FE model, as shown in Fig. 1a. To simulate conventional water immersion ultrasonic testing, the models of pressure acoustics (water) and solid mechanics (TC4) in the COMSOL Multiphysics FE software is used.

Ultrasonic waves are the mechanical vibration in the form of fluctuations in an elastic medium. The linear equation of ultrasonic field in isotropic solid medium is as Eq. (10)^37^:

10 $$ \rho \frac{{\partial^{2} u}}{{\partial t^{2} }} = \left( {\lambda + 2\mu } \right)\nabla \nabla u - \mu \nabla \times \nabla \times u $$

where *ρ* is the density of the medium, *u* is the particle displacement, *λ* and *μ* are the lame constant.

To simulate ultrasonic waves using the FE method, it is necessary to first divide the computational domain into a certain number of mesh cells according to accuracy and efficiency. Since the pore diameter *d* and the pore spacing distance *g* are far smaller than the wavelength *λ*_TC4_ of ultrasonic wave in titanium alloy, it is important to divide the model into different meshing regions to simplify the calculation. As shown in Fig. 1b, the free triangular mesh method is used for mesh division, and the maximum element size is used to constrain the mesh cells. Outside the diffusion bonding area, the maximum element size is set to be 1/8 of the ultrasonic wavelength in the current medium. Inside the diffusion bonding area, to accurately calculate the response of microscopic pores to ultrasonic waves, the maximum element size is set to be 1/8 of the smaller value between *d* and *g*.

After mesh division, the displacement interpolation function is constructed, and the motion equation of the system is derived from Eq. (10) as Eq. (11)^38^:

11 $$ Q(t) = Ma(t) + Sv(t) $$

where *a*(*t*) is the systematic acceleration vector, *v*(*t*) is the node velocity vector, *Q*(*t*) is the node load vector, *M* is the systematic mass matrix, and *S* is the stiffness matrix.

The material parameters of TC4 in the COMSOL FE model in this paper are as follows: density *ρ* = 4500 kg/m^3^, longitudinal wave velocity *c*_L_ = 5900 m/s, and shear wave velocity *c*_S_ = 3050 m/s.

The ultrasonic transducer is excited with the normal displacement at the set position on the water layer boundary. The load is a three-period sinusoidal oscillation signal modulated by the Hanning window, expressed as Eq. (12)^39,40^:

12 $$ {\text{signal}}(t) = ( - 1)^{c} \times (1 - \cos (2\pi f_{0} t/c)) \times \sin (2\pi f_{0} t) \times {\text{win}}(t) $$

where *c* is the pulse period, *f*_0_ is the center frequency of the ultrasonic transducer, and win(*t*) is the *c* periodic rectangular waves at the frequency *f*_0_. According to the FE model, the propagation process of ultrasonic waves corresponding to every time series is obtained, as shown in Fig. 1c. The subsequent calculation and analysis are based on the echo signal of diffusion bonding defects extracted from the A-scan signal in Fig. 1d.

### Reliability of transducers for phase calculation

**Figure 2:**
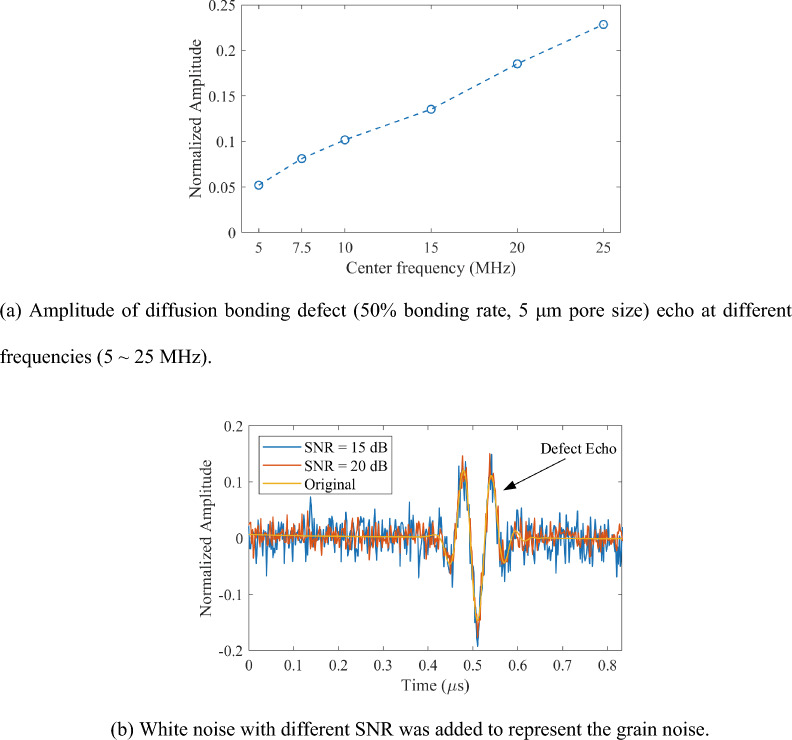
The amplitude of defect at different frequencies and the added noise to defect echo.

It is clear that, to use interfacial stiffness *ĸ* to evaluate the quality of diffusion bonding, a certain amplitude of the interface echo is required. Figure 2a shows the echo amplitude of the diffusion bonding defect (with a bonding rate of 50% and a pore size of 5 μm) at transducer center frequency of 5–25 MHz. The reference signal of the titanium-water interface echo at the corresponding frequency is used for normalization. As the testing frequency increases, the echo amplitude increases for the diffusion bonding defects of same parameter. The calculation of interfacial stiffness *ĸ* depends on the true phase measurement of the defects echo signal. As the center frequency of the transducer increases, the noise immunity (grain structure noise, electrical noise, etc.) decreases^41–43^. The true phase measurement of the signal is sensitive to noise. Therefore, Gaussian white noise is used with different signal-to-noise ratio (SNR) to the echo signal of diffusion bonding defect obtained from the simulation to investigate the reliability of different ultrasonic testing frequency in true phase calculation. The SNR of the defect echo signal with noise is defined as follows:

13 $$ {\text{SNR}} = 20\lg \frac{{A_{{{\text{signal}}}} }}{{A_{{{\text{noise}}}} }}{ = }10\lg \frac{{P_{{{\text{signal}}}} }}{{P_{{{\text{noise}}}} }}\left( {{\text{dB}}} \right) $$

where *A*_signal_ is the amplitude of the diffusion bonding defect signal, and* A*_noise_ is that of noise; *P*_signal_ is the power of the defect echo signal, and *P*_noise_ is that of noise. By calculating the value of added noise power *P*_noise_ under the corresponding SNR, the Gaussian white noise of corresponding power can be added to the simulation signal. The defect echo signals after adding different SNR noises to represent the grain noise are shown in Fig. 2b.

**Figure 3:**
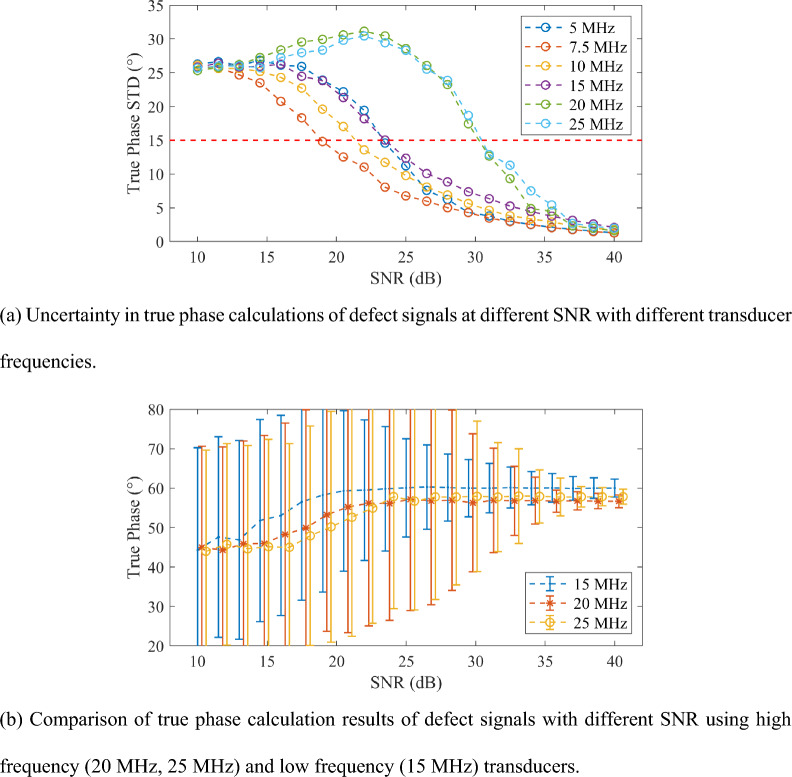
True phase calculation for echo signal of diffusion bonding defect (50% bonding rate, 5 μm pore size) at different transducer frequencies.

The standard deviation of the true phase calculations of several uncorrelated sets of signals with the same SNR can reflect the reliability of the true phase calculations under different transducer frequencies. As shown in Fig. 3a, the true phase measurement standard deviations of noise echo signals with SNR 10–40 dB with six sets of transducers at 5–25 MHz are calculated. The standard deviation is calculated from 1000 uncorrelated signals at the corresponding SNR. As shown in Fig. 3a, with the increase of the SNR, the uncertainty of true phase calculation (standard deviation) using transducers of 5–15 MHz decreases gradually. In contrast, the calculation uncertainty using transducers of 20 MHz and 25 MHz decreases gradually at the SNR larger than of 25 dB. When the standard deviation of true phase calculation is smaller than 15°, the transducer can measure the true phase of the signal under the corresponding SNR. Due to the wide echo pulse width of the 5 MHz transducer, the diffusion bonding defect echo is partially superimposed on the bottom echo, resulting in a higher SNR than those using transducers of higher frequency (7.5 MHz and 10 MHz) when the calculation uncertainty reaches 15°^34^. To reach the threshold of phase measurement uncertainty (15°), transducers with higher frequency, 20 MHz and 25 MHz, require an SNR of 32 dB; while the 15 MHz transducer only needs an SNR of 25 dB.

Figure 3b shows a comparison of the phase calculation reliability using the transducers with center frequency of 15, 20 and 25 MHz. The error bars are the true phase calculation uncertainty corresponding to the standard deviation calculated from 1000 uncorrelated echo signals at the corresponding SNR. Each data point corresponds to the average value of the above 1000 calculated true phase. A shorter error bar represents a smaller uncertainty of the frequency at the current SNR and a higher reliability. Figure 3b shows that the true phase calculation uncertainty of the 15 MHz transducer is significantly better than that of the 20 MHz and 25 MHz transducers when the SNR is lower than 35 dB. It shows that the calculation reliability of the 15 MHz transducer is better than those of the 20 MHz and 25 MHz transducers. The ultrasonic waves with higher frequency have higher sensitivity to micro voids such as diffusion bonding defects^13^. Therefore, considering both the sensitivity and reliability, transducer with a center frequency of 15 MHz is recommended for diffusion bonding defect testing.

**Figure 4:**
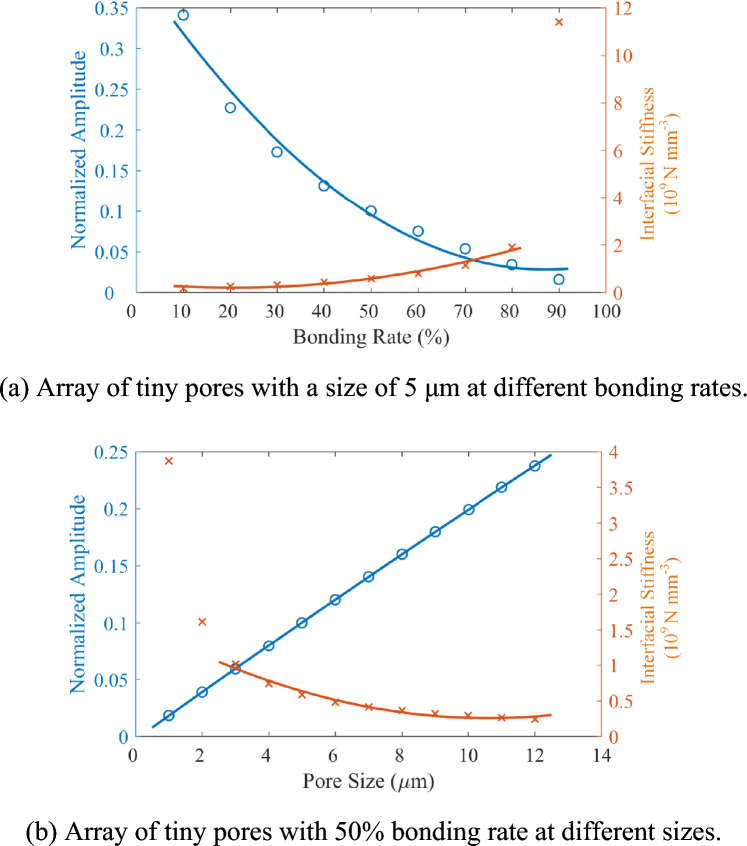
The echo amplitude and interfacial stiffness *ĸ* of the tiny pores in diffusion bonding interface with different specifications.

The ultrasonic response of diffusion bonding defects with different bonding rate and pore size is calculated using a transducer central frequency of 15 MHz. The defect echoes are extracted. To further calculate the phase value variation corresponding to each defect echo, the diffusion bonding interface is set as the titanium-water interface. The echo signal of this interface is calculated and extracted as the reference signal. The responses of the diffusion bonding defect are calculated for the pore of 5 μm with the bonding rate of 10% ~ 90%. The defect echoes are extracted, and the corresponding interfacial stiffness *ĸ* is calculated based on Eq. (5), as shown in Fig. 4a. With the increase of bonding rate, the amplitude of defect echo decreases while the interfacial stiffness *ĸ* increases. At the bonding rate of 90%, the defect echo amplitude is excessively weak, resulting in a significant mutation in the interfacial stiffness *ĸ*. It can be seen from Fig. 4a that when the bonding rate is 10–80% at the pore size of 5 μm, the spring model-based interfacial stiffness *ĸ* can effectively characterize the diffusion bonding quality.

In addition, the responses of diffusion bonding defects with a pore size of 1–12 μm at the bonding rate of 50% are calculated. The corresponding interfacial stiffness *ĸ* is also calculated, as shown in Fig. 4b. As the pore size increases, the amplitude of the defect echo increases while the interfacial stiffness *ĸ* decreases. When the pore size is 1 μm and 2 μm, the weak defect echo leads to a significant mutation in the stiffness *ĸ*. It can be seen from Fig. 4b that when the pore size is 3–12 μm at the bonding rate of 50%, the interfacial stiffness *ĸ* based on the spring model can also reflect the diffusion bonding quality.

### Ultrasonic testing model for complex surface

**Figure 5:**
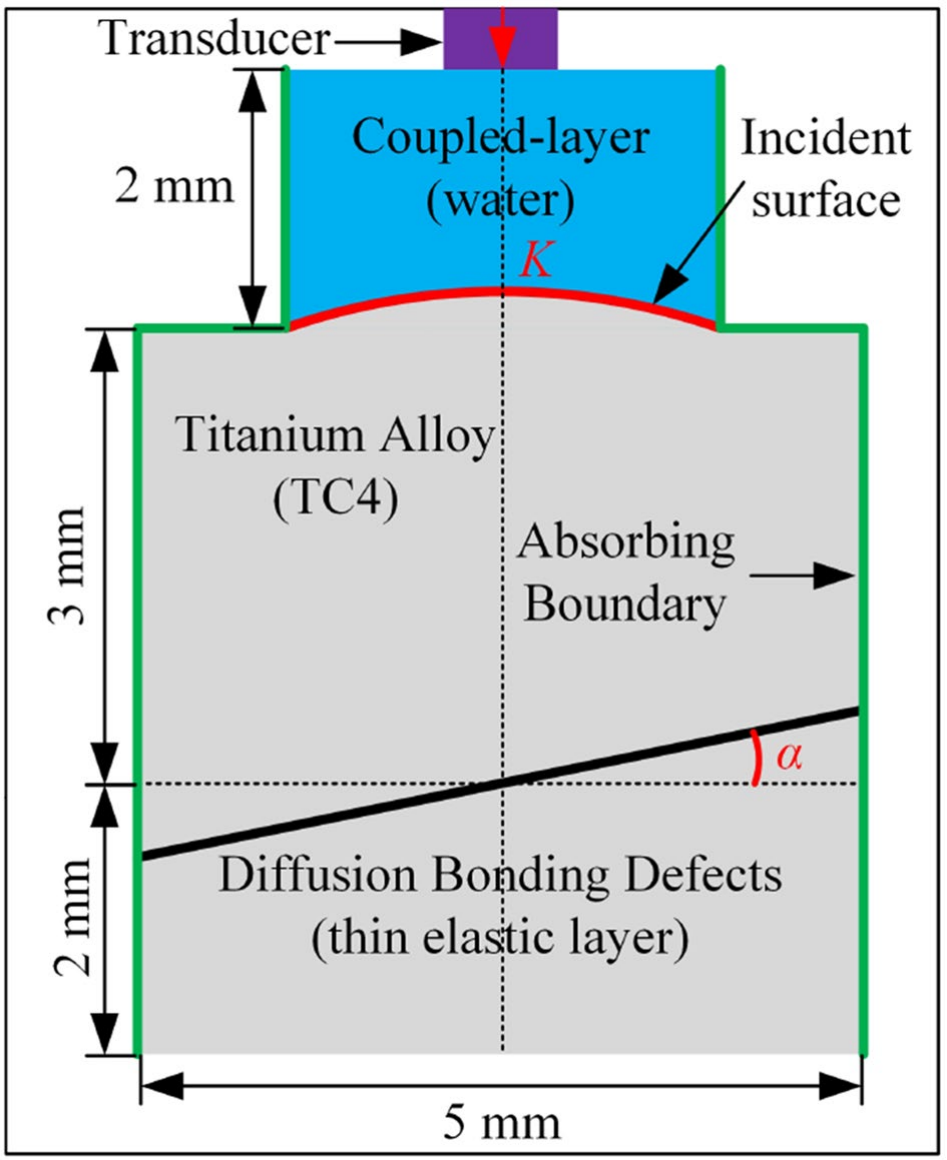
FE model considering the curvature *Κ* of the incident surface and the inclination angle *α* of the diffusion bonding interface.

In the water immersion ultrasonic testing of diffusion bonding in complex-surface components, there are mainly problems such as the curvature effect of the incident surface, the error caused by inclination angle, and the varying depth of the diffusion bonding interface. The testing mechanism for the diffusion bonding interface under a complex surface is simplified as a two-dimensional cross-section schematic shown in Fig. 5. The influence of curvature of incident surface and inclination angle of diffusion bonding interface on the true phase calculation of the defect echo signal is investigated based on corresponding FE model. To simplify the calculation, a thin elastic layer is used instead of a tiny pores array to characterize diffusion bonding defects in this model.

**Figure 6:**
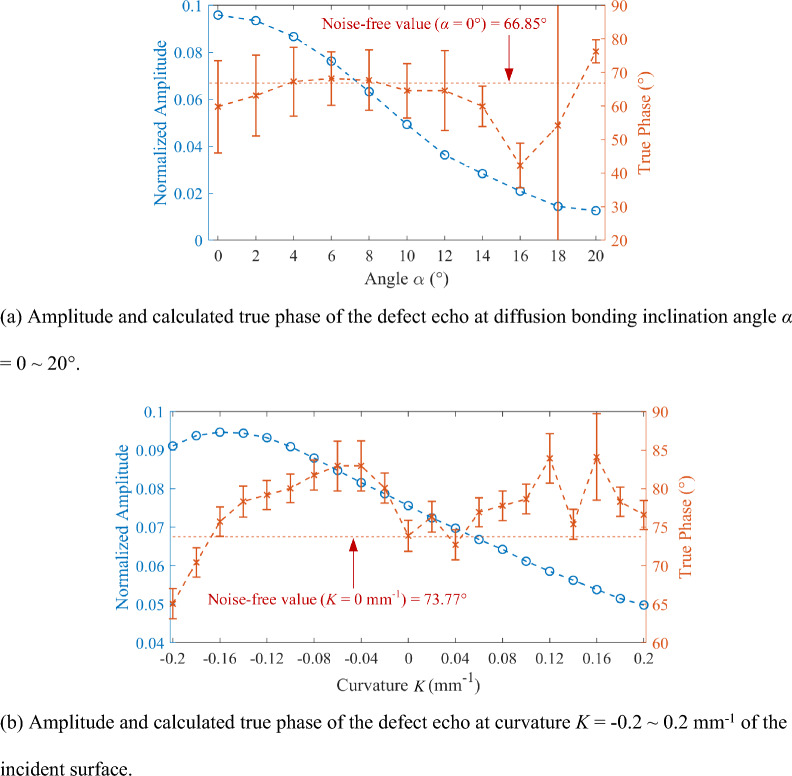
The simulation results for diffusion bonding defects echo measurement of the FE model considering the complex incident surface.

Figure 6a shows the simulation of the defect echo amplitude corresponding to the true phase measurement of a 15 MHz transducer at an inclination angle of 0°–20°. Error bars correspond to the standard deviation of the true phase calculated from 1000 uncorrelated echo signals at a SNR of 30 dB. Each data point corresponds to the average value of the above 1000 calculated true phase. The value of the true phase corresponding to the defect echo without noise is 66.85° when the inclination angle is 0. As shown in Fig. 6a, the defect echo signals are affected by noise and inclination angle. The calculated value of the true phase fluctuates within a range of 10° from 66.85° as the inclination angle increases from 0 to 14°, and the standard deviation of the true phase is lower than the threshold of 15°. When the inclination angle reaches 16°, the defect echo diverges due to the large angle, leading to the serious deviation of the true phase calculation value, and the calculation result is not reliable. Therefore, the results of the true phase calculation can be within the acceptable error range (threshold of 15°) at inclination angles no larger than 14°.

The amplitude and true phase of the defect echo using the 15 MHz transducer at the incident surface curvature of −0.2 to 0.2 mm^−1^ are calculated as shown in Fig. 6b. Error bars correspond to the standard deviation of the true phase calculated from 1000 uncorrelated echo signals at a SNR of 40 dB. Each data point corresponds to the average value of the above 1000 calculated true phase. The value of the true phase corresponding to the defect echo without noise in this model is 73.77° when the curvature is 0 mm^−1^. As shown in Fig. 6b, the results of the true phase calculations can also be within the acceptable error range (threshold of 15°), except for few calculated points. The significant error points may result from the incomplete absorption of the interface echoes by the absorbing layer of the FE model. According to the above simulation analysis, the true phase calculation is valid within a specific inclination angle range (no larger than 14°). Therefore, when the transducer is placed along the normal direction, the influence of the curvature of the incident surface on the true phase measurement can be ignored.

### Static ultrasonic field model

The varying depth of the diffusion bonding interface in complex-surface components makes it difficult to detect diffusion bonding defects. Due to the limit of mechanical accuracy and the motion control, the ultrasonic C-scan of complex surfaces usually uses a fixed water length. Therefore, the ultrasonic field distribution of the conventional water immersion focusing transducer used is consistent. To ensure the quality of the echo signals of diffusion bonding, the ultrasonic field focal spot area of the transducer is required to cover the bonding area. Based on the characteristics of varying diffusion bonding depth, a static ultrasonic field computation model is established to select a suitable focusing transducer for diffusion bonding interface detection of a complex-surface component.

**Figure 7:**
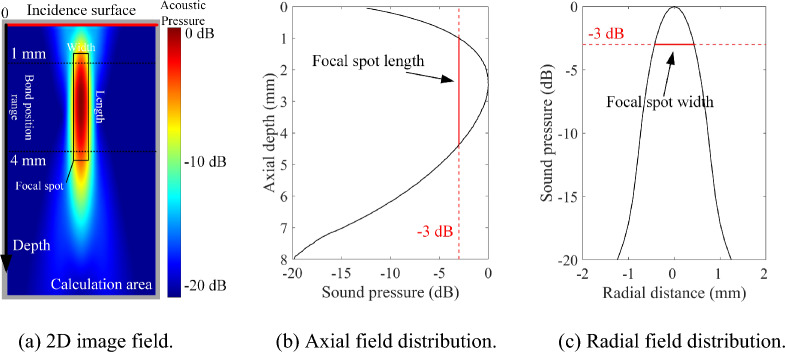
The coverage range of the focal spot in the ultrasonic field of the ultrasonic transducer.

As shown in Fig. 7, the ultrasonic fields of transducers with different parameters in TC4 titanium alloy are simulated^44^. By evaluating the focal spot size (length and width), parameters (center frequency and crystal diameter) of the transducer suitable for the detection of diffusion bonding at a variable depth are determined. The depth between the diffusion bonding interface and the incident surface of ultrasonic waves in the TC4 component with complex surface ranges in 1–4 mm. To maximum focal coverage, the transducer is focused at a depth of 2.5 mm from the surface, as shown in Fig. 7a. The focal length of the transducer is limited to 50.3 mm considering practical conditions. The longitudinal wave velocity of the material is measured by a TC4 Titanium alloy standard test block. The key parameters of the ultrasonic field computation model are shown in Table 1.

**Table 1:** Parameters of the computation model for the static field of the ultrasonic transducers.

Frequency (MHz)	Piezoelectric crystal diameter (mm)	Focal length (mm)	Water path (mm)	Longitudinal wave velocity (m/s)	Focal spot threshold (dB)
5–25	6–30	50.3	41	5900	−3

**Figure 8:**
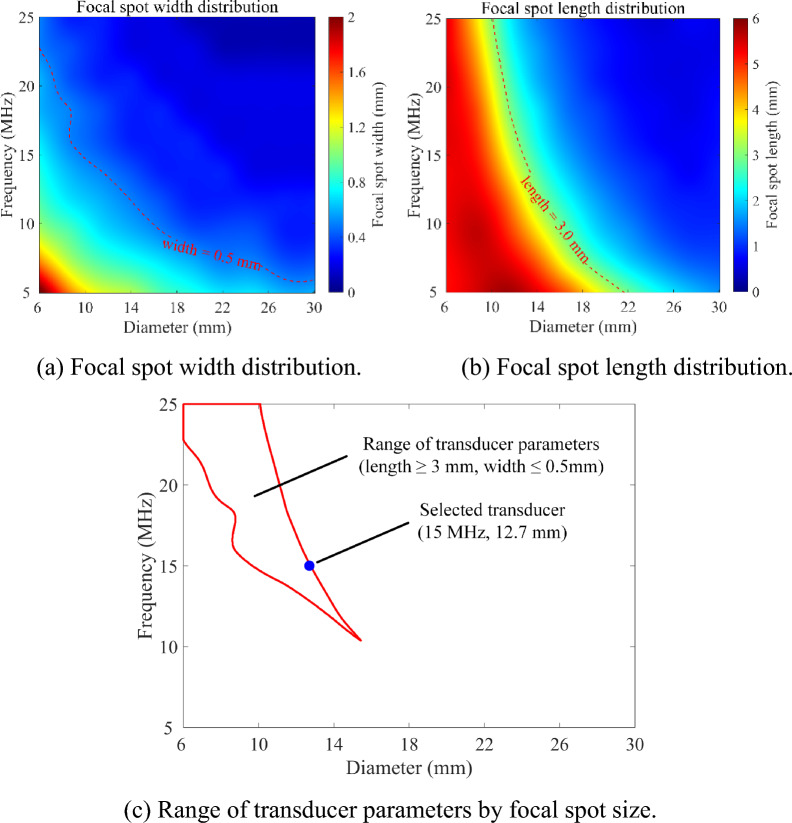
Simulated results of focal spot size under different diameter and frequency parameters.

Considering the practical situation of the adapter of the experimental equipment and the manufacture of the transducer, the static ultrasonic fields under a large number of transducer parameters are calculated in the frequency range of 5–25 MHz, and the crystal diameter range of 6–30 mm. The calculation methods for the focal spot width and length are shown in Fig. 7b, c, respectively. With a focal spot threshold of −3 dB, the calculated focal spot size distribution is shown in Fig. 8a, b. As shown in Fig. 7a, the focal length of the transducer needs to reach 3 mm to ensure that the focal spot of the transducer covers the bonding area. To ensure the transverse resolution of an ultrasonic transducer, the focal width is limited to less than 0.5 mm. The range of transducer parameters that satisfies the limit to focal size is shown in Fig. 8c. Within this range, an focusing transducer with a frequency of 15 MHz, and a crystal diameter of 12.7 mm is selected for the further diffusion bonding detection experiment of the complex-surface component.

## Experiments and analysis

**Figure 9:**
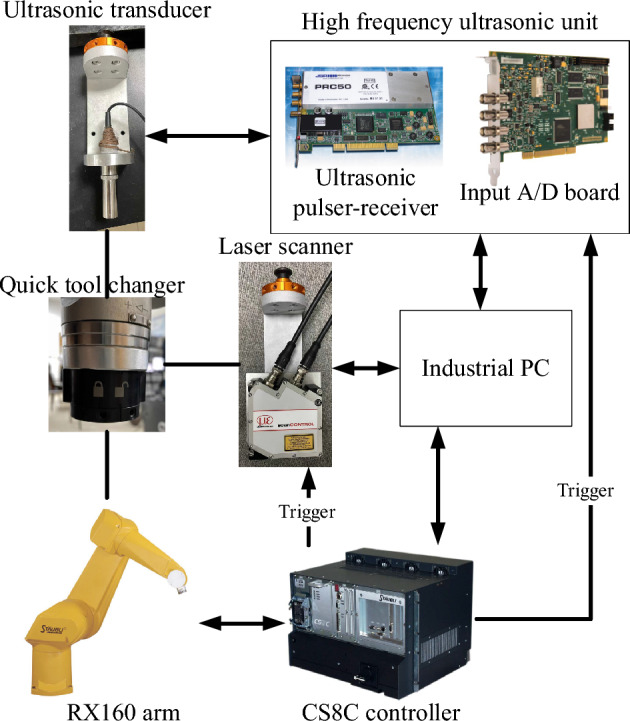
Ultrasonic testing experiment system.

The schematic of a self-developed ultrasonic testing experiment system is shown in Fig. 9. The RX-160 six-axis robot arm is used as the scanning structure, supporting the ultrasonic transducer and the laser profile scanner. The laser profiling module uses an LLT2900 linear laser profile scanner. The high-frequency ultrasonic system consists of a PRC50 ultrasonic card and an AL12260 data acquisition board. The experiment system has the functions of laser profiling and ultrasonic scanning trajectory planning for a complex-surface component. Based on the simulation results in "Ultrasonic testing model for complex surface", a focusing immersion transducer manufactured by GE with a central frequency of 15 MHz, a crystal diameter of 12.7 mm, and a focal length of 50 mm is selected for the experiment.

**Figure 10:**
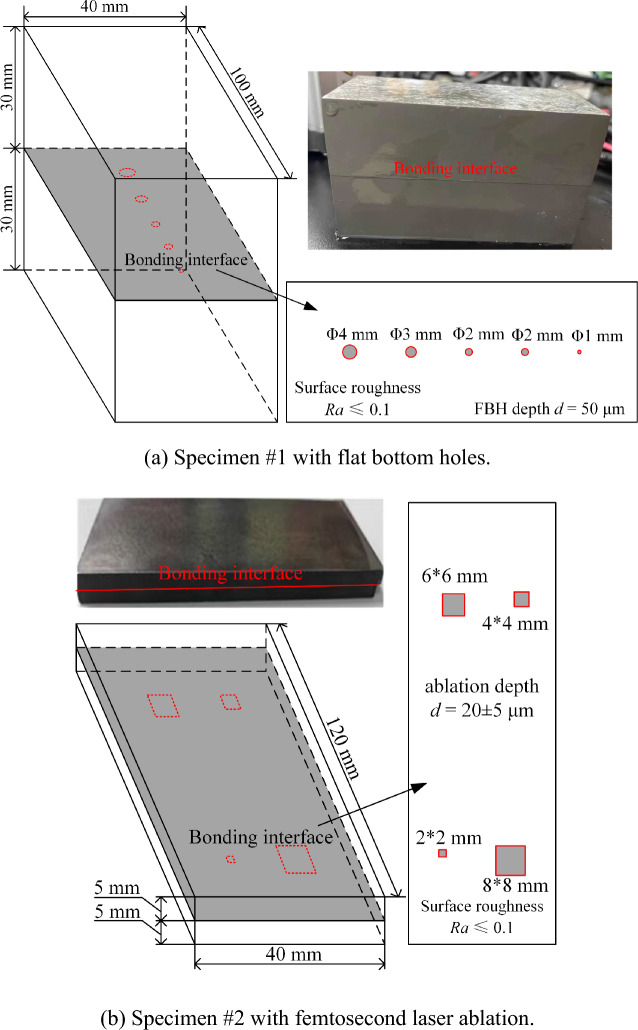
Defects embedment before the bonding of TC4 test specimens.

Two TC4 titanium alloy plate specimens with different thicknesses of embedded artificial defects are diffusion bonded via a vacuum diffusion bonding process for 90 min at a temperature of 920 ℃, and a pressure of 3 MPa. The artificial diffusion bonding interface defects are prepared by pre-treatment of the bonding surface. As shown in Fig. 10a, a series of flat bottom holes with a depth of 50 μm and a diameter of 1 to 4 mm are machined on the bonding surface of specimen #1. Part of the bonding surface of the thin-plate TC4 specimen #2 is ablated by femtosecond laser^45^ with a theoretical depth of 20 ± 5 μm, as shown in Fig. 10b. To ensure the bonding quality, the bonding surfaces with embedded defects are finely polished and pickled before bonding.

Based on the analysis of the phase measurement capability of transducers at different frequencies in "Ultrasonic response model of diffusion bonding defects", 15 MHz is selected as the center frequency to test the diffusion bonding interface. For different depths of the diffusion bonding interface, a 15 MHz transducer with a focal length of 205 mm is selected for specimen #1 in the ultrasonic C-scan experiments while a 15 MHz transducer with a focal length of 50.3 mm is used for specimen #2. To focus the ultrasonic field of the transducer on the diffusion bonding interface, the water path *P* should be calculated according to Eq. (14):

14 $$ P = F - \frac{{c_{{\text{L}}} }}{{c_{{\text{w}}} }}d $$

where *F* is the focal length of the transducer; the longitudinal wave velocity of TC4, *c*_L_, is 5900 m/s; the longitudinal wave velocity of water,* c*_w_, is 1483 m/s; and *d* is the depth of the diffusion bonding interface. According to Eq. (14), the water path of specimen #1 and specimen #2 are set to be 80 mm and 30 mm, respectively. The scanning step is 0.2 mm for both specimens.

**Figure 11:**
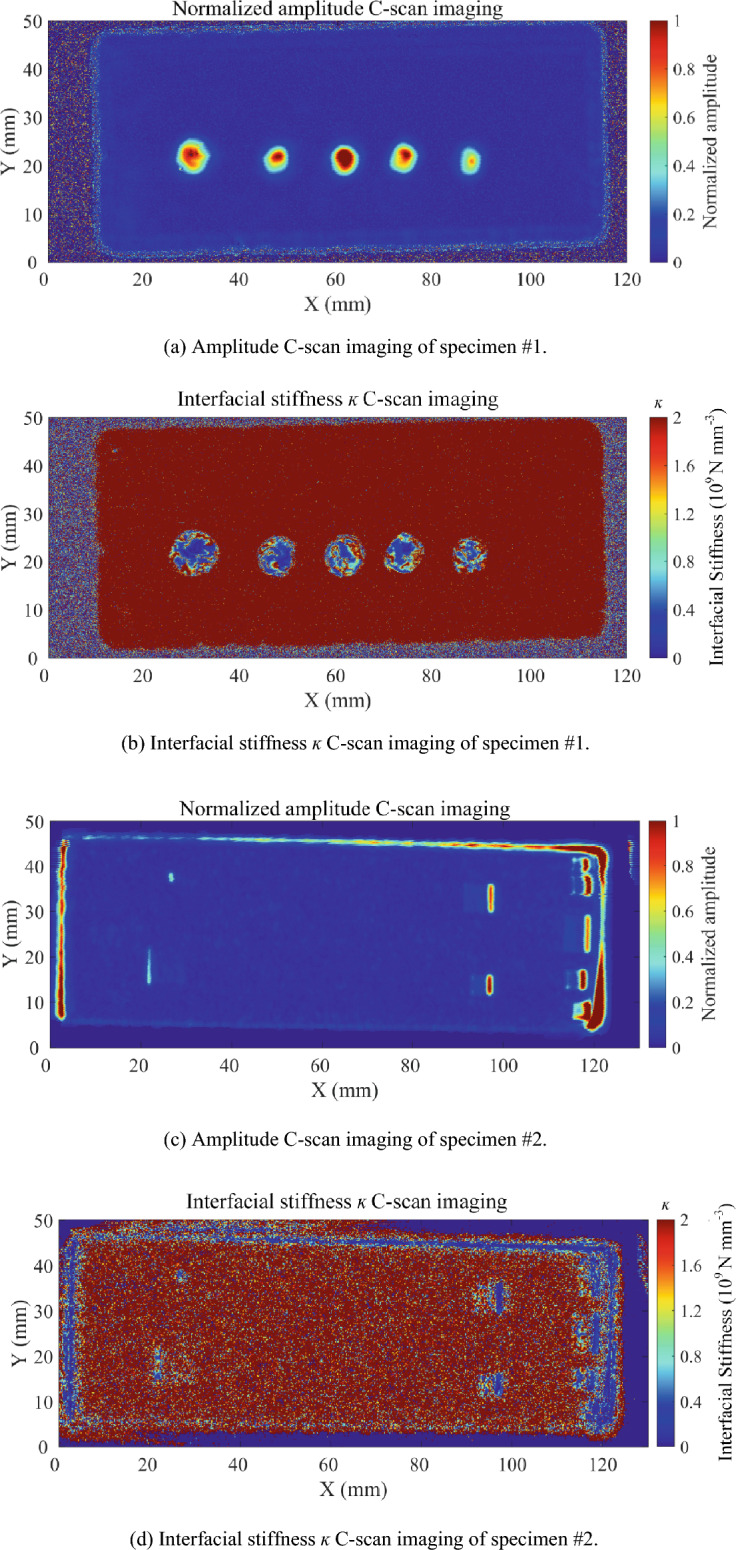
Testing results of specimen #1 and specimen #2 at diffusion bonding interface (when the normalized amplitude of the interface signal is ≤ 0.05, it can be regarded as well bonded).

The amplitude imaging results of the signal gate at the diffusion bonding interface are shown in Fig. 11a, c. The amplitudes of all points in the above images are normalized by the maximum absolute value of the signal at the interface position. All embedded defects in specimen #1 are detected with strong echo amplitudes. This results from that the flat bottom holes with a depth of 50 μm did not fuse after bonding, while holes with large longitudinal sizes were formed on the diffusion bonding interface.

In terms of specimen #2, distinct echo signals can be obtained at one side of the defects (not fixed); otherwise, the echo signals are weak. The echo signals from the edge positions of specimen #2 in the C-scan imaging are caused by the dislocation of the upper and lower plates during bonding and not involved in the quality evaluation of the bonded interface. Since the theoretical ablation depth of the titanium alloy surface is 20 μm in the initial state of the diffusion bonding, after diffusion bonding, micro-void-like defects with a diameter smaller than 20 μm are distributed at the bonding interface. The defect diameter is far smaller than the half wavelength of the ultrasonic wave. Therefore, the echoes of the ablation area of specimen #2 are generally weak. Due to the characteristics of the femtosecond laser, the error between the actual ablation depth and the theoretical value can be about 5 μm. Thus, the echo amplitude in the defect-embedded region is not uniform.

Based on the collected full-wave data and the reference signals of the titanium-water interface collected before bonding, the interfacial stiffness *ĸ* can be calculated by Eq. (5) and used for evaluation. The testing results based on the interfacial stiffness *ĸ* of specimens #1 and #2 are shown in Fig. 11b, d. When the normalized amplitude of the echo at the bonding interface was lower than 0.05, it can be regarded as a good bonding point, and the *ĸ* value was set to infinity. The interfacial stiffness* ĸ* calculated from the interface echo can reflect the bonding quality of the diffusion bonding interface. The interface stiffness around the defect area is lower than that in the well-bonded area. Embedded defects in specimens #1 and #2 can be detected in the interfacial stiffness *ĸ* C-scan images based on the interface spring model. The surface of specimen #2 to be bonded is ablated by femtosecond laser to a depth of about 20 μm. After diffusion bonding, the fabricated defects are closer to the actual situation than the artificial defects in specimen #1. The echo signals of defects are weak, so it is important to set a higher signal receiving analog gain in the ultrasonic unit to improve the received echo energy. The analog gain is set to be 45 dB for specimen #1 and 60 dB for specimen #2. It should be noticed that due to the high analog gain in the detection of small defects in specimen #2, a lot of noise is introduced, so there is much more noise in Fig. 11d and a lower SNR compared with Fig. 11b.

**Figure 12:**
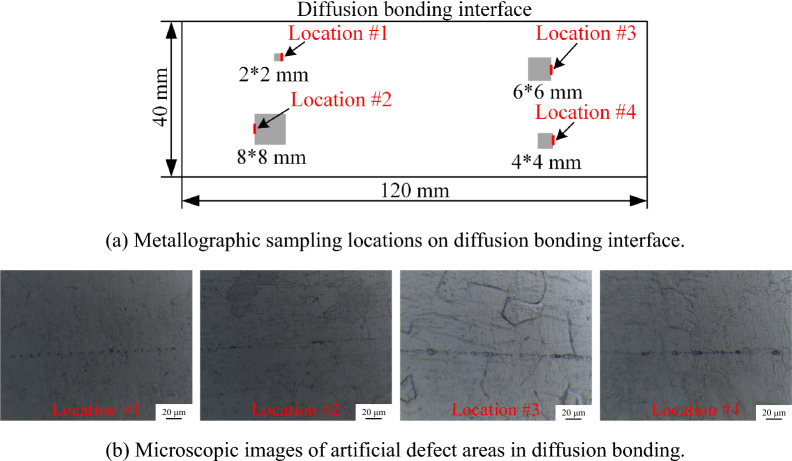
Microscope metallography images of the bonding interface at four locations of the embedded defects in Specimen #2.

As shown in Fig. 12a, four pre-embedded defect regions of specimen #2 are sampled, and the defect morphology of the bonding interface is observed by a microscope. The micro images of diffusion bonding interface at the four sampling locations are shown in Fig. 12b. All four locations have micrometer-sized void defects ranging from 3 to 5 μm along the bonding lines. Due to the misalignment during the bonding of specimen #2, it is difficult to accurately match the metallography at the sampling position with the ultrasonic signal in the C-scan image. The metallographic image of the bond cannot be used to study the relationship between the ultrasonic echo signal of the interface and the size and distribution of diffusion bonding defects, but can only be used to suggest the existence of diffusion bonding defects at these locations.

**Figure 13:**
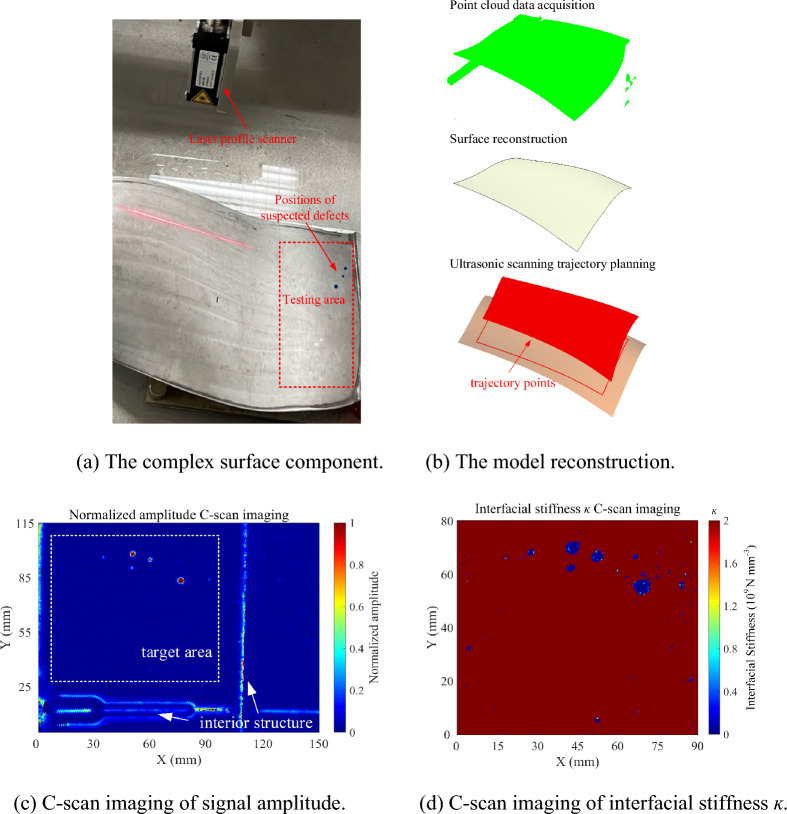
Testing results of a complex surface component at diffusion bonding interface.

The complex-surface component is shown in Fig. 13a, which is detected by ultrasonic C-scan with the parameters used in "Ultrasonic testing model for complex surface". The process of testing surface reconstruction and scanning trajectory planning is shown in Fig. 13b. The normalized amplitude C-scan image of the gate at the diffusion bonding interface is shown in Fig. 13c. The internal structure echoes of the detected object are not involved in the evaluation of the diffusion bonding interface quality. The titanium-water interface signal corresponding to the depth of the bond is used as the reference signal. The interfacial stiffness *ĸ* of the diffusion bonding interface in the target area of Fig. 13c is calculated by Eq. (5). As shown in Fig. 13d, the distribution of suspected defects in the diffusion bonding interface can also be observed. The blue points in Fig. 13a are the positions of internal diffusion bonding defects inferred from the suspected defect signals in the C-scan image. Due to the slight change of bond depth in the calculated area of interfacial stiffness *ĸ* (within 0.3 mm), only one depth of the titanium-water interface echo is used as the reference signal for the diffusion bonding interface, which also introduced a significant error. In practical applications, to reduce the reference signal error, a C-scan experiment should be performed on a complex-surface component before diffusion bonding. The echo signals of the water-titanium interface corresponding to the depth of each point in the bonding interface should be collected as the reference signals for subsequent calculation of interface stiffness* ĸ* for each point.

Comparing Fig. 13c, d, it can be found that the interfacial stiffness *ĸ* imaging has higher sensitivity to weak defect signals compared to the conventional amplitude imaging. The interfacial stiffness *ĸ* takes into account both the true phase and amplitude information of the defect echo, and theoretically has stronger ability to detect small defects than the conventional amplitude method. At the same time, high sensitivity also means that it is susceptible to noise and cause false detection. In Fig. 11, the defect size distortion in the interfacial stiffness *ĸ* imaging may be caused by noise interference. The denoising method based on the signal processing needs further research.

## Conclusions

In this paper, an interfacial stiffness characterization method has been proposed to test the defect on the diffusion bonding interface of complex surface components. The corresponding FE models of ultrasonic response and the model of static ultrasonic field have been established considering the characteristics of the complex surface and the diffusion bonding defects. The ultrasonic responses of diffusion bonding defects, the influence of complex surface factors, and the optimization of transducer parameters are simulated and analyzed. The effectiveness of the proposed testing method using interfacial stiffness *ĸ* based on the spring model for diffusion bonding defects has been verified with the experimental results. The following conclusions can be drawn:

1. According to the reliability analysis of the true phase calculation for the diffusion bonding defects echo signals containing noise, 15 MHz is recommended as the center frequency of the transducer for the TC4 titanium alloy components. According to the evaluation of the focal spot size, the parameters range diagram is proposed to recommend transducers covering the diffusion bonding area.
2. The simulation echo interfacial stiffness *ĸ* values of diffusion bonding defects with different bonding rate of 10–90% and pore size of 1–12 μm are calculated. The *ĸ* value is positively correlated with the bonding rate and negatively correlated with the pore size. The correlation between the interfacial stiffness *ĸ* and the bonding quality of the diffusion bonding interface is verified.
3. The true phase calculation error of the diffusion bonding defect echo below inclination angle of 14° is within the range below 15° when the ultrasonic wave is incident vertically.
4. In microscope metallography images of the cross-section samplings of an embedded defect specimen, the void defects of 3–5 μm distributed along the diffusion bonding interface has been found, and their positions were consistent with the ultrasonic testing image.
5. The interfacial stiffness *ĸ* imaging for the diffusion bonding interface of the complex surface component shows that compared with the conventional amplitude imaging, the interfacial stiffness *ĸ* method has higher sensitivity to the weak diffusion bonding defect signals. Meanwhile, it also has the limitations of reduced anti-noise ability, which leads to false detection and quantitative size distortion.

## Data Availability

The datasets generated and/or analyzed during the current study are available from the corresponding author on reasonable request.

## List of symbols

*ĸ*: Interfacial stiffness
*R*_*i*_: Reflection coefficient of interface *i*
*Z*_*i*_: Acoustic impedance of material *i*
*m*: Mass per unit area
*ω*: Angular frequency
*Z*: Harmonic mean acoustic impedance
*η*: Relative acoustic impedance mismatch
Φ_*i*_: Phase shift of interface *i*
*f*: Frequency of transducer
*H*_*i*_(*f*): Amplitude of signal *i*
*φ*(*ω*): Phase spectrum
*ω*_0_: Center angular frequency
*φ*_*i*_: True phase of signal *i*
*β*: Slope of fitting-line
*r*: Bonding rate
*g*: Spacing distance in pore array
*d*: Pore diameter
*ρ*: Density of the medium
*u*: Particle displacement
*λ*: Lame constant
*μ*: Lame constant
*λ*_*i*_: Wavelength in material *i*
*a*(*t*): Systematic acceleration vector
*v*(*t*): Node velocity vector
*Q*(*t*): Node load vector
*M*: Systematic mass matrix
*S*: Stiffness matrix
*f*_0_: Center frequency of transducer
win(*t*): Rectangular waves
*A*_*i*_: Amplitude of echo *i*
*P*_*i*_: Power of echo *i*
*K*: Curvature of incident surface
*α*: Inclination angle of diffusion bonding interface

## Abbreviations

NDT: Non-destructive testing
FE: Finite element
SNR: Signal-to-noise ratio
